# EGF/EGFR upregulates and cooperates with Netrin-4 to protect glioblastoma cells from DNA damage-induced senescence

**DOI:** 10.1186/s12885-018-5056-4

**Published:** 2018-12-04

**Authors:** Li Li, Yulun Huang, Yuge Gao, Tengfei Shi, Yunyun Xu, Huini Li, Marko Hyytiäinen, Jorma Keski-Oja, Qiuying Jiang, Yizhou Hu, Zhimin Du

**Affiliations:** 1 Department of Oncology, the Second Clinical College, Harbin Medical University, Harbin, People’s Republic of China; 2 Department of Neurosurgery and Brain and Nerve Research Laboratory, the First Affiliated Hospital of Soochow University, Suzhou, China; 3 Institute of Pediatrics, Children’s Hospital of Soochow University, Suzhou, China; 4 Departments of Virology and Pathology, Faculty of Medicine, the Haartman Institute, Translational Cancer Biology Research Program and Helsinki University Hospital, University of Helsinki, Helsinki, Finland; 5 Department of pharmacy, the Second Clinical College, Harbin Medical University, Harbin, People’s Republic of China; 6 Present address: Division of Molecular Neurobiology, Department of Medical Biochemistry and Biophysics, Karolinska Institute, Stockholm, Sweden

**Keywords:** Epidermal growth factor, Netrin-4, Glioblastoma, Cell senescence

## Abstract

**Background:**

Glioblastoma multiforme (GBM) is the most malignant central nervous system tumor. Alkylating agent, temozolomide (TMZ), is currently the first-line chemotherapeutic agent for GBM. However, the sensitivity of GBM cells to TMZ is affected by many factors. And, several clinic trials, including co-administration of TMZ with other drugs, have failed in successful treatment of GBM. We have previously reported that Netrin-4 (NTN4), a laminin-like axon guidance protein, plays a protective role in GBM cell senescence upon TMZ-triggered DNA damage. However, the master regulator of NTN4 needs further elucidation. Epidermal growth factor/Epidermal growth factor receptor (EGF/EGFR) can modulate the expression of various extracellular matrix related molecules, and prevent DNA damage in GBM cells. In this study, we investigated the relationship between EGF/EGFR signaling and NTN4, and explored their effect on therapeutic efficacy in GBM cells upon TMZ treatment.

**Methods:**

Co-expression analysis were performed by using the RNA sequencing data from NIH 934 cell lines and from single cell RNA sequencing data of GBM tumor. The co-expressing genes were used for GO enrichment and signaling pathway enrichment. mRNA expression of the target genes were quantified by qPCR, and cell senescence were investigated by Senescence-Associated Beta-Galactosidase Staining. Protein phosphorylation were observed and analyzed by immunoblotting. The RNA sequencing data and clinical information of TMZ treated patients were extracted from TCGA-glioblastoma project, and then used for Kaplan-Meier survival analysis.

**Results:**

Analysis of RNA sequencing data revealed a potential co-expression relationship between *NTN4* and *EGFR*. GO enrichment of *EGFR*-correlated genes indicated that EGFR regulates GBM cells in a manner similar to that in central nervous system development and neural cell differentiation. Pathway analysis suggested that *EGFR* and its related genes contribute to cell adhesion, extracellular matrix (ECM) organization and caspase related signaling. We also show that EGF stimulates NTN4 expression in GBM cells and cooperates with NTN4 to attenuate GBM cell senescence induced by DNA damage, possibly via AKT and ERK. Clinical analysis showed that co-expression of EGFR and NTN4 significantly predicts poor survival in TMZ-treated GBM patients.

**Conclusions:**

This study indicates that *EGF/EGFR* regulates and cooperates with NTN4 in DNA damage resistance in GBM. Therefore, our findings provide a potential therapeutic target for GBM.

## Background

Glioblastoma multiforme (GBM) is the most common malignant tumor of the central nervous system [1, 2]. Even with treatment, the median survival time of glioblastoma patients following diagnosis is 12 ~ 15 months [3, 4]. Alkylating agent, temozolomide (TMZ), is currently the first-line chemotherapeutic agent for the treatment of glioblastoma. The therapeutic action of TMZ is exerted by triggering DNA damage, which induces GBM cell apoptosis and senescence [5]. However, even with adjuvant TMZ chemotherapy, the 2-year overall survival rate of GBM patients only reaches ~ 27% [6–8]. The therapeutic efficacy of TMZ depends on self-repair of DNA damage [6], the rescue effect from RTK signaling [9–11], and cell remodeling in the microenvironment [12].

Our previous study showed that Netrin-4 (NTN4) protects glioblastoma cells from TMZ-induced senescence through rescuing the phosphorylation of AKT and ERK [13]. NTN4 was originally identified as a secreted laminin-like axon guidance protein in neuronal development [14, 15]. In addition to the central nervous system, NTN4 is broadly expressed in non-neural cells, and regulates tissue development and tumor progression by modulating various cellular functions [16–23]. In glioblastoma, NTN4 is highly expressed at the tumor invading edge [24] and promotes glioblastoma cell proliferation via ITGB4-Akt signaling [25]. Although the expression of netrin-associated receptors including ITGA6 and ITGB4, is regulated by various growth factors, especially by Epidermal growth factor*/*Epidermal growth factor receptor EGF/EGFR [26–29], the upstream regulators of NTN4 expression and of GBM cell senescence remain largely unknown.

EGF is a growth factor that stimulates cell growth, proliferation, and differentiation [30]. EGF binds to EGFR family receptors with high affinity. This stimulates ligand-induced dimerization, activating the intrinsic protein-tyrosine kinase activity of the receptor. The tyrosine kinase activity, in turn, initiates signal transduction cascades that results in a variety of biochemical changes within the cell: a rise in intracellular calcium levels, increased glycolysis and protein synthesis, and increases in the expression of certain genes including the gene for EGFR, that lead to the regulation of various biological functions [31, 32]. EGF/EGFR signaling can protect cells from DNA damage through different downstream signaling pathways [33–35]. However, clinical trials of EGF/EGFR inhibitors failed to show any therapeutic efficacy, even in combination with TMZ [36, 37]. Therefore, upon induction of DNA damage in GBM cells, co-inhibition of RTK signaling and the extracellular matrix remodeling, may provide a new strategy for GBM treatment. Here, using experimental and bioinformatic analyses, we describe EGF/EGFR upregulation of NTN4 expression and EGF/EGFR cooperation with NTN4 to attenuate GBM cell senescence induced by DNA damage.

## Methods

Immunoblot analysis, immunofluorescence, transfection of cells, total RNA extraction, reverse transcription, and real-time reverse transcription-PCR were performed as described [13, 25]. Senescence-associated beta-galactosidase staining and bioinformatic analysis were performed as below.

### Cell lines and reagents

U251MG cells (Health Science Research Resources Bank, Osaka, Japan) were cultured according to the supplier’s instructions. Primary GBM cells were isolated according to previous description [38, 39], and then cultured in in Dulbecco’s modified essential culture medium (DMEM) supplied with 10% heat inactivated fetal calf serum (FCS) (Gibco, USA), 100 IU/ml penicillin, 50 mg/ml streptomycin and 1% L-glutamine.. In this study, all experiments have obtained patients’ consent and been approved by the Ethic Committee for Harbin Medical University. The following primary antibodies and recombinant protein were used: anti-NTN4 from R&D systems; anti-phospho-p44/43MAPK (ERK1/2, Thr202/Tyr204), anti-phospho-mammalian target of rapamycin (mTOR; Ser2448), anti-phospho-SRC(Ser416), and anti-phospho-AKT (Ser473) from Cell Signaling Tech. (Danvers, MA, USA); anti-β-tubulin from Santa Cruz Biotechnology (Santa Cruz, CA; recombinant NTN4 from R&D Systems (Minneapolis, MN, USA).

### H_2_O_2_ and EGF treatment

H_2_O_2_ (Merck KGaA, Germany) treatment was performed on > 90% confluent cells (~ 1 × 10^5^ cells per well in 6-well plates) to avoid variability of H_2_O_2_ toxicity as described in [40]. Cells were incubated in 2 ml/well culture medium containing 150 μM H_2_O_2_ for 48 h at 37 °C in a 5% CO_2_ atmosphere, and then split to 5000 cells per well in 24-well plates in fresh DMEM.

EGF (Sigma E9644) treatment was performed at a concentration of 50 ng/ml, and was added to growing cells in 0.05% serum DMEM in 24-well plates every 2 days. Subsequently, the cells were gently washed twice with PBS, and then cultured under 10% serum fresh DMEM for further experiments.

### Transfection of cells

Cells were cultured in six-well plates to 50 to 80% confluence and transfected using 2 μg plasmid DNA and 6 μl FuGENE 6 transfection reagent for each transfection according to the manufacturer’s instructions (Boehringer Mannheim, Mannheim, Germany). Stably transfected cells were selected with ∼0.8 mg/ml G418 (Gibco/Invitrogen) in complete medium. The expression levels of each indicated gene in transfected cells were analyzed by Q-RT-PCR.

### Senescence-associated Beta-galactosidase staining (SA β-gal staining)

Cells were cultured in 24-well plates at densities of 3000~ 5000 cells per well for the indicated times. Subsequently, the cells were washed once with PBS and then fixed in PBS containing 0.5% glutaraldehyde at room temperature for 20 min. After two PBS washes, the cells were incubated with SA β-gal substrate solution (1 mg/ml X-gal, 40 mM citric acid-sodium phosphate buffer, pH 6.0, containing 5 mM potassium ferricyanide, 5 mM potassium ferrocyanide, 150 mM NaCl, and 2 mM MgCl_2_) at 37 °C in the dark for 16–20 h [40]. The reaction was terminated by removing SA β-gal substrate solution and washing twice with PBS. The cells were stored in 70% glycerol at 4 °C. The cells were photographed using an Axiovert 200 inverted epifluorescence microscope (Carl Zeiss). The ratio of blue-stained cells was analyzed with ImageJ (National Institutes of Health, Bethesda, MD).

### Bioinformatic analysis

Co-expression analysis was performed using Spearman’s rank correlation coefficient between the expression of EGFR and all other genes, in the dimension of all single cells. Distance Correlation analysis of gene expression was described previously [41–43]. Briefly, for each gene, pairwise Euclidean distances were generated in the dimension of all cells. After scaling the values by mean, the co-variance of EGFR and other genes was calculated upon the distance matrix between EGFR and all other genes. GO enrichment was assessed using Python Package jdrudolph/goenrich with modification on the enrichment of GO bioprocess, and the pathway enrichment was analyzed by Python Package PyPathway. Lifelines Package was used for Kaplan-Meier survival analysis. 3D axes plots and Violin plots were generated by Matplotlib and Seaborn.

### Statistical analysis

All numerical values represent the mean ± SE or SD. Statistical significance was determined with the nonparametric Mann-Whitney U test.

## Results

### EGF stimulates Netrin-4 expression in U251MG cells

**Fig. 1:**
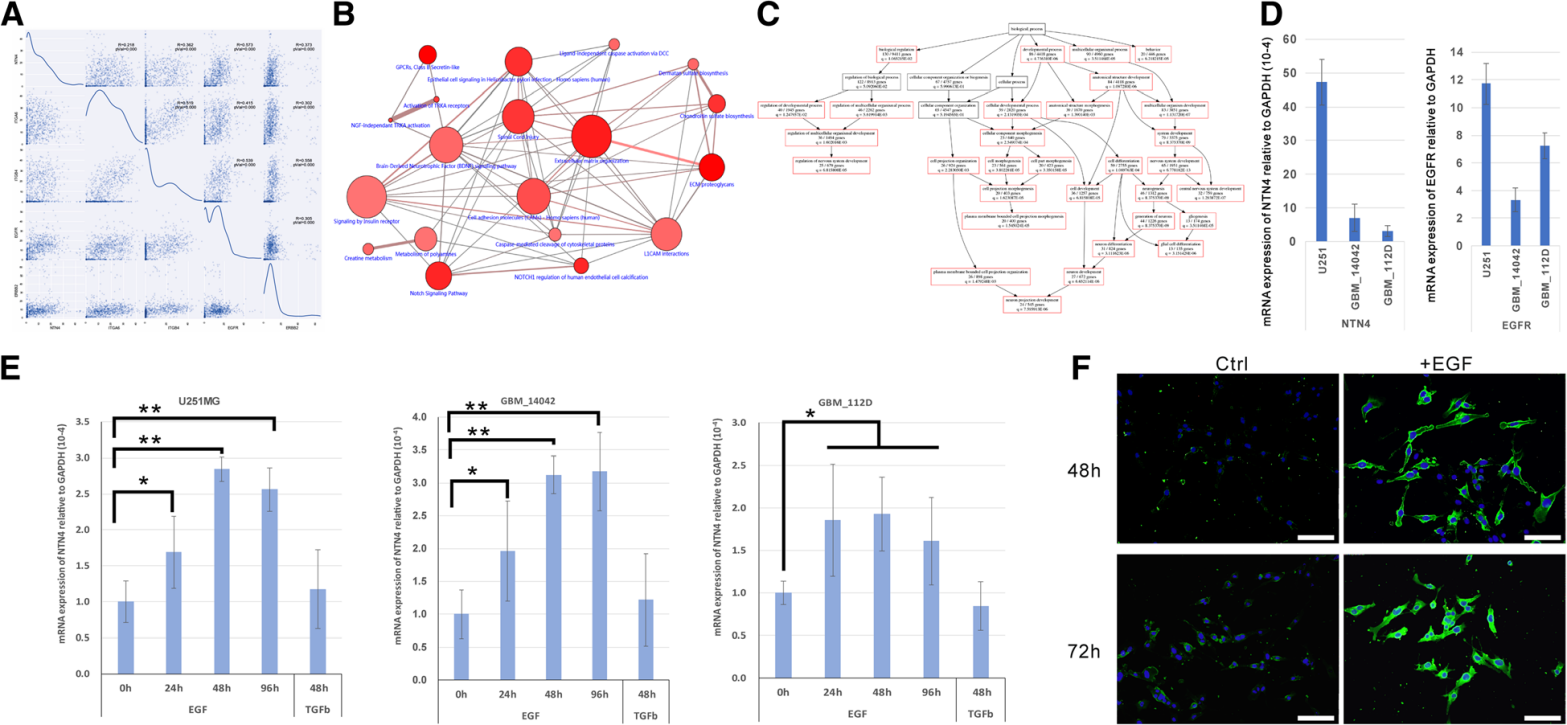
EGF contributes to NTN4 expression in GBM cells. **a** On the basis of RNA sequencing data from 934 NIH cell lines, we calculated the Spearman product-moment correlation coefficient. The co-expression analysis revealed that the expression of EGFR correlates with NTN4 (R_value = 0.573) and ITGB4 (R_value = 0.539). **b** & **c** According to scRNA sequencing data of glioblastoma, 191 genes co-expressed with EGFR were enriched by overlapping Spearman product-moment correlation coefficient and distant correlation analysis. These 191 genes were further used for signaling pathway enrichment and GO bioprocess enrichment. ECM organization and Caspase-related signaling pathways were enriched by signaling pathway analysis (**b**). Nervous system development and glial cell differentiation were enriched by GO bioprocess analysis (**c**). **d** Using Q-RT-PCR, the mRNA levels of NTN4 and EGFR were determined in two primary glioblastoma cell lines: GBM-14042 and GBM-112D. **e**-**f** EGF treatment was performed at the concentration of 20 ng/ml and was re-applied every 48 h. 24 h, 48 h, and 96 h after EGF treatment, the mRNA levels of NTN4 were determined in U251MG, GBM-14042 and GBM-112D cells by Q-RT-PCR (**e**). After treatment for 48 h and 96 h, the expression of NTN4 was 2 to 3-fold higher than control group. The enhancing effects of EGF on NTN4 expression (green) were also detected by immunofluorescence in U251MG at 48 h and 72 h, and the Dapi staining was shown in blue (**f**)

ITGA6, ITGB4 are regulated by different growth factors [20, 27, 44–47]. Therefore, as a potential ligand of laminin integrins, NTN4, is possibly expressed in response to stimulation by growth factors. By analyzing RNA sequencing data of NIH 934 human cell lines [48], we explored the co-expression relationships among NTN4, ITGA6, ITGB4, EGFR, and ErBB2 in these cell lines. We found that expression of NTN4 significantly correlated with EGFR (Fig. 1a). Meanwhile, we confirmed that the expression of EGFR is tightly linked to that of ITGA6 and ITGB4. These findings are in line with our previous observation that NTN4/ITGB4 protect GBM cells from TMZ-induced cell senescence, and indicate that NTN4 expression is possibly monitored by the EGF signaling pathway. To further understand the downstream pathway and biological functions of EGF signaling in glioblastoma, we extracted single-cell RNA sequencing (scRNAseq) data of grade IV glioblastoma [49]. After overlapping the results from Spearman correlation coefficient and Distance (pDist) Correlation analyses, we obtained a list of 191 genes that significantly correlate with EGFR, including NTN4 (SI Table I). Using these 191 genes, we further enriched for GO bioprocess and signaling pathways underlying EGFR signaling (SI Table II). Cell adhesions, ECM organization and Caspase-related signaling pathways were the top effectors of EGFR expression (Fig. 1b). Nervous system development and glial cell differentiation were the top GO bio-processes of EGFR-related genes (Fig. 1c). To validate this observation, U251MG GBM cells and two primary GBM cell lines, GBM-14042 and GBM-112D cells, were treated with EGF for 24 h, 48 h, and 96 h. U251MG express the mRNA of both EGFR and NTN4 at decent level, but does not express MGMT [13, 50], and the similar expression profile were detected in both GBM-14042 and GBM-112D cells by qRT-PCR (Fig. 1d). In addition, both of GBM-14042 and GBM-112D cells do not express MGMT (negative data, not shown here). Furthermore, we observed that NTN4 expression were significantly increased upon EGF stimulation at both mRNA level (Fig. 1e) and protein level (Fig. 1f).

The GO function and signaling pathway enrichment revealed that EGFR might contribute to cell differentiation, central nervous system development, ECM re-organization, and netrin/DCC related caspase activation. Meanwhile, EGF stimulation upregulates the expression of NTN4. Thus, using a senescence assay induced by H_2_O_2_ and TMZ, we observed the relationship between EGF stimulation/NTN4 expression and GBM cell senescence.

### NTN4 and EGF protect GBM cells from DNA damage-induced senescence

**Fig. 2:**
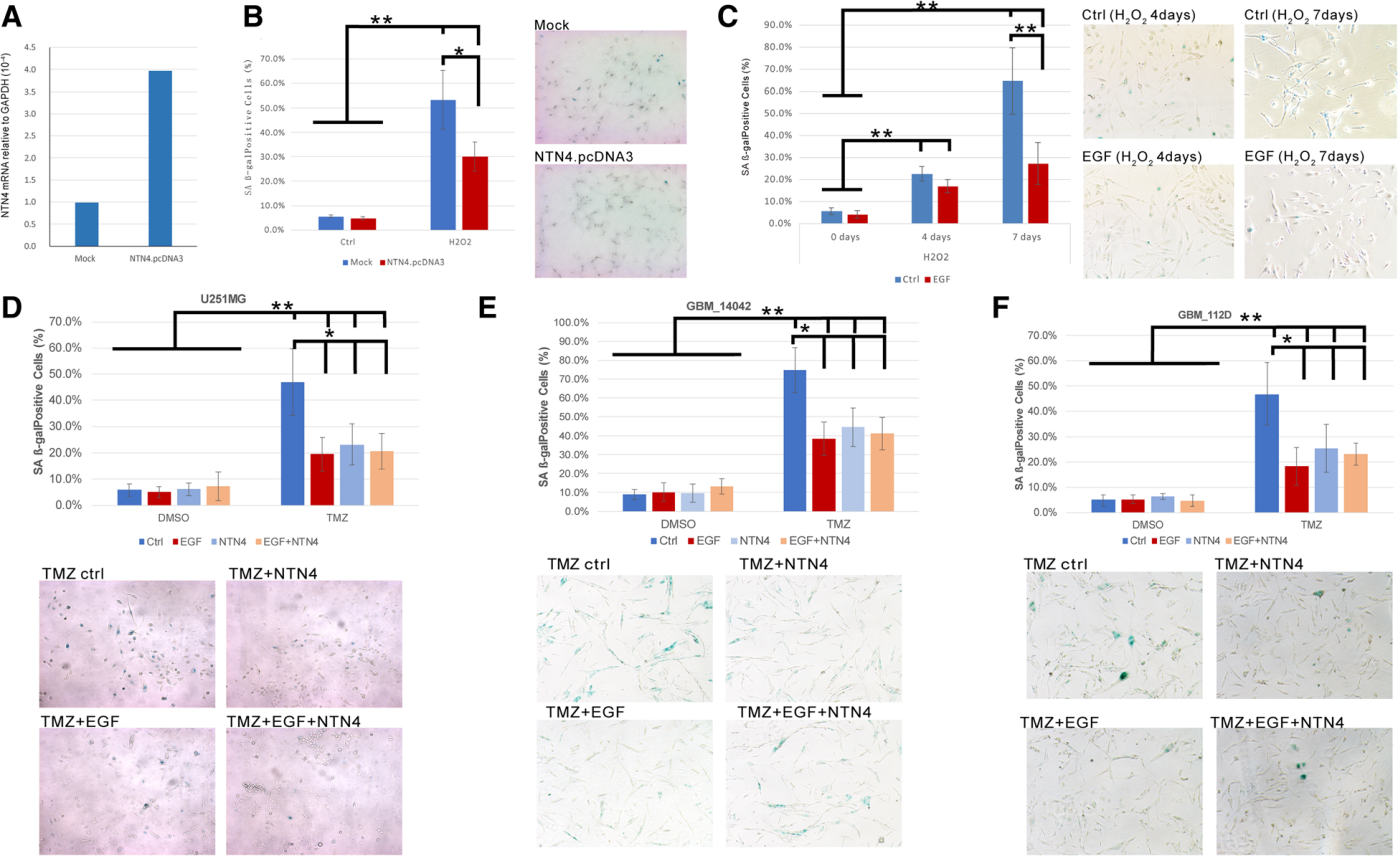
EGF and NTN4 delayed cell senescence induced by H_2_O_2_ and TMZ. (**a**) The expression levels of NTN4 were determined by Q-RT-PCR in U251MG cells overexpressing NTN4 (NTN4.pcDNA3) and in mock control U251MG cells (Mock). (**b**) Five days after H_2_O_2_ treatment, mock cells and NTN4.pcDNA3 cells were fixed and analyzed by beta-gal staining. H_2_O_2_ triggered senescence of U251MG cells, while NTN4 overexpression significantly decreased the number of senescent cells induced by H_2_O_2_. (**c**) After the H_2_O_2_ treatment, U251MG cells were treated with EGF (40 ng/mL) every two days. On the 4th and 7th day after EGF treatment, U251MG cells were fixed and analyzed by the beta-gal staining. Four days after EGF treatment, the number of senescent U251MG cells was significantly less compared with control cells. Seven days after EGF treatment, the number of senescent U251MG cells was ~ 2-fold less than in EGF non-treated groups. (**d-f**) After TMZ treatment, U251MG cells were treated with EGF (40 ng/mL), NTN4 (50 ng/ml), and EGF + NTN4, separately. All the proteins were re-supplied every two days. After treatment for 4 days, TMZ strongly induced senescence in U251MG cells (**d**), GBM-14042 (**e**) and GBM-112D cells (**f**), while EGF, NTN4, and EGF + NTN4 attenuated the pro-senescence effect of TMZ. Mean ± SE, *n* ≥ 3, * *p*-value < 0.05, ** p-value < 0.01

We have previously demonstrated that NTN4 contributes to the resistance of TMZ-triggered GBM cell senescence [13]. Hydrogen peroxide can also induce cell senescence by triggering DNA single- and double-strand breaks. Here we used a H_2_O_2_-induced senescence assay to further understand the effect of NTN4 and EGF on GBM cell senescence. We constructed U251MG cells expressing NTN4 (Fig. 2a). We then detected the number of senescent cells after H_2_O_2_ treatment using a senescence-associated beta-galactosidase assay. We observed significantly fewer senescent NTN4-overexpressing cells compared with mock control cells (Fig. 2b). Thus, NTN4 overexpression delayed U251MG cell senescence induced by H_2_O_2_. We then detected whether EGF could reduce the number of senescent U251MG cells induced by H_2_O_2_. On the 4th day after H_2_O_2_ treatment, we observed less senescence in U251MG cells treated with EGF compared with non-EGF treated cells. On the 7th day after H_2_O_2_ treatment, the number of senescent cells in the EGF treatment group was significantly less than that in the non-treatment control group. (Fig. 2c). There was a significant difference in U251MG senescence between cells treated and not treated with EGF for 7 days. With EGF (40 ng/ml) treatment, senescence of U251MG cells induced by H_2_O_2_ decreased. The concentration of 40 ng/ml EGF inhibited U251MG cell senescence efficiently.

To reconfirm the effect of NTN4 and EGF on GBM cell senescence induced by DNA damage, we treated U251MG cells, GBM-14042 and GBM-112D cells with TMZ to induce cell senescence. TMZ-treated cells were co-administrated NTN4, EGF, or combined NTN4/EGF, and then senescent cells were quantified. Both EGF and NTN4 treatment delayed cell senescence compared with non-treatment control cells, while the combined NTN4/EGF treatment did not provide any additive effect on rescuing cell senescence (Fig. 2d-f). Therefore, either EGF or NTN4 can attenuate GBM cell senescence triggered by DNA damage, and they may recruit similar pathways in response to DNA damage.

### The EGF/NTN4 signaling axis contributes to the phosphorylation of Src, ERK and AKT

**Fig. 3:**
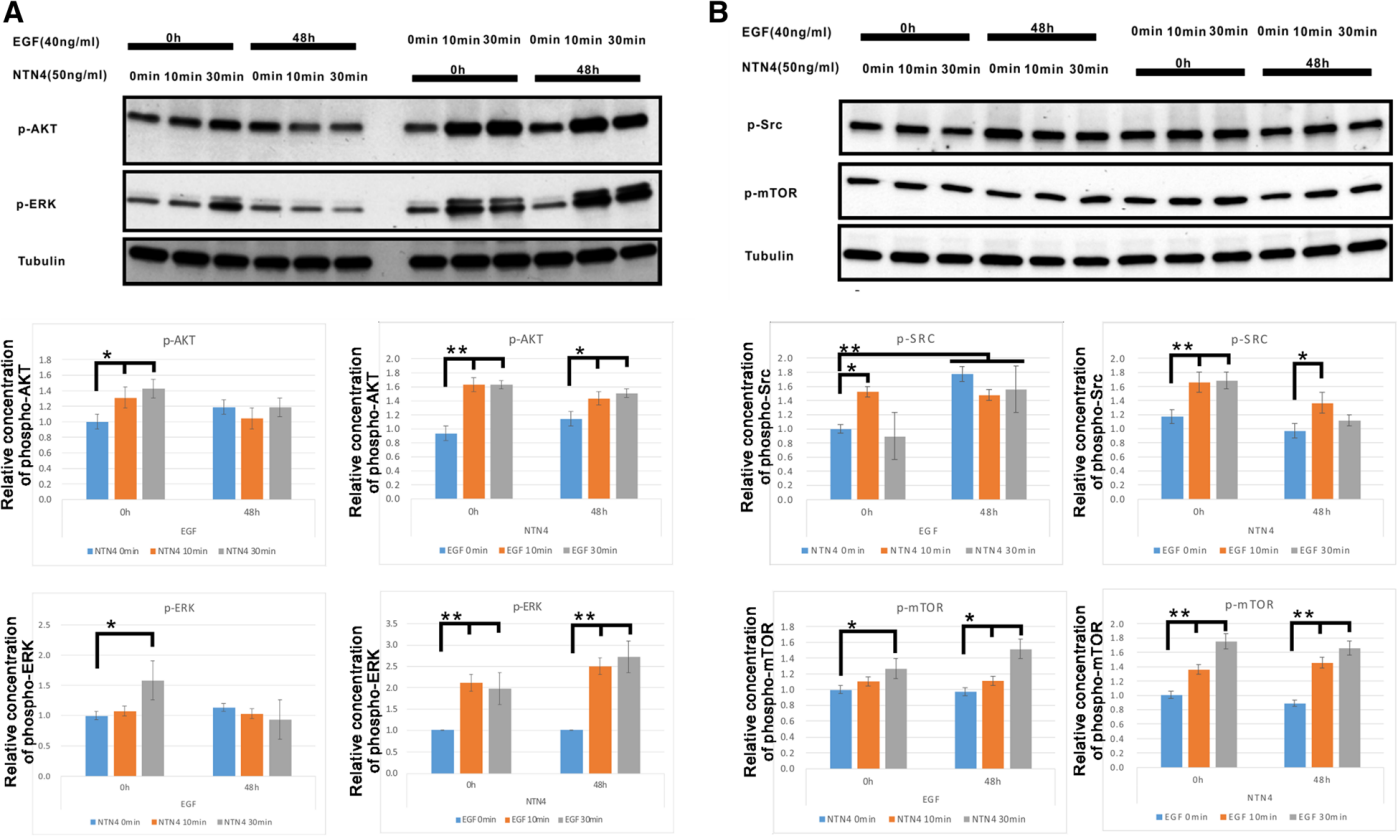
EGF regulates NTN4 to stimulate the phosphorylation of AKT, ERK, and Src. U251MG cells were treated with EGF (40 ng/mL) and NTN4 (50 ng/ml) for 48 h. Then these cells were treated with NTN4 (50 ng/mL) or EGF (40 ng/mL) for 10 min or 30 min. Subsequently, cells were lysed and the phosphorylation of AKT, ERK, Src and mTOR detected. **a** Short term stimulation of EGF significantly enhanced AKT phosphorylation, and a similar phenomenon was observed after 48 h of stimulation with recombinant NTN4. Addition of exogenous NTN4 increased p-AKT levels at 10 min and 30 min. After EGF stimulation for 48 h, NTN4 was not able to further increase p-AKT. Long term addition of exogenous NTN4 did not affect p-ERK. However, short term stimulation of EGF significantly enhanced ERK phosphorylation, regardless of long term NTN4 stimulation. Addition of exogenous NTN4 increased levels of p-ERK at 30 min. p-ERK did not show any significant change after 48 h of stimulation with EGF. **b** Long term addition of exogenous NTN4 did not affect p-Src. Stimulation with EGF for 10 min significantly increased p-Src, with or without 48 h of stimulation with exogenous NTN4. Stimulation with EGF for 30 min enhanced p-Src, which was abolished after treatment with NTN4 for 48 h. Addition of exogenous NTN4 increased p-ERK after 10 min. Although p-Src was strongly up-regulated after 48 h of treatment with EGF, NTN4 did not additionally stimulate Src phosphorylation. Long term addition of exogenous NTN4 did not affect p-mTOR. EGF significantly increased p-mTOR levels at both 10 min and 30 min, with or without 48 h stimulation with exogenous NTN4. Addition of exogenous NTN4 significantly enhanced p-mTOR at 30 min. EGF treatment for 48 h did not change p-mTOR levels; however, p-mTOR levels were strongly increased when combined with NTN4 stimulation for 30 min. Mean ± SE, n ≥ 3, * *p*-value < 0.05, ** *p*-value < 0.01

We then explored the combined effect of EGF and NTN4 on four major downstream signaling pathways in U251MG cells: ERK, AKT, Src and mTOR (Fig. 3). EGF treatment for 48 h slightly but not significantly increased p-ERK, p-AKT levels, and strongly upregulated Src phosphorylation. Short term EGF stimulation with or without NTN4 treatment for 48 h can enhance p-ERK, p-AKT, p-Src, and p-mTOR levels. Treatment with NTN4 for 48 h did not significantly alter any signaling pathways. Short term treatment of wild type U251MG cells with NTN4 increased p-ERK, p-AKT, and p-Src levels, while this effect was covered by long term stimulation with EGF. Interestingly, short-term NTN4 treatment had a continuously stimulatory effect on p-mTOR, even after 48 h EGF treatment. Thus, the EGF/NTN4 axis may contribute to the phosphorylation of ERK, AKT, and Src. Meanwhile, NTN4 itself may have a separate effect on mTOR phosphorylation.

### Co-expression of EGFR/NTN4 predicts poor survival in TMZ-treated GBM patients

**Fig. 4:**
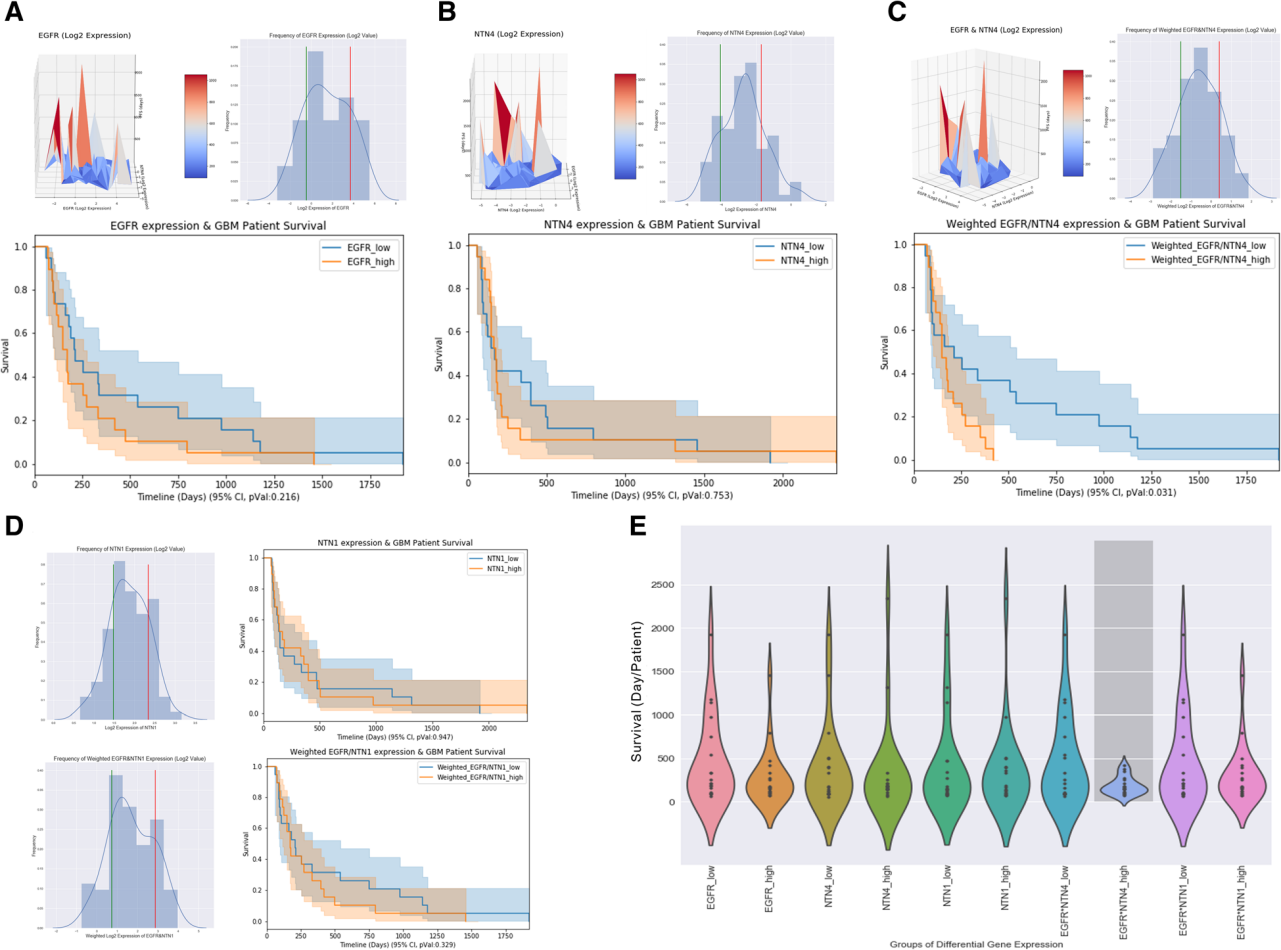
The co-expression of EGFR and NTN4 contribute to TMZ resistance in GBM patients. Clinical information and gene expression data of totally 92 TMZ-treated GBM patients were extracted from the TCGA-glioblastoma dataset. To describe the contribution of EGFR and NTN4 to survival time of TMZ-treated patients, we used both 3D axes surface plots and Kaplan-Meier analysis. In 3D axes surface plots, the X and Y axes represent the expression of EGFR and NTN4, respectively. The Z axis represents survival time of each patient. Here, we selected the 20% of patients (top 19 patients) with either the highest (right side of red line) or lowest (left side of green line) expression of target genes and compared the survival time. **a** High EGFR expression slightly but not significantly predicts poor patient survival. **b** The expression of NTN4 itself does not correlate with patient survival. **c** Weighted high expression of EGFR and NTN4 significantly predicts poor patient survival. **d** NTN1 was used as a control in this study. Neither single NTN1 expression nor weighted expression of EGFR and NTN1 correlated with patient survival. **e** Violin plot representing the relationship between all different combinations of EGFR/NTN4/NTN1 expression and survival time of TMZ-treated GBM patients. Dark grey bar-box indicates a significant p-value (< 0.05). Only when both EGFR and NTN4 were highly expressed in patients, were survival times lowest in TMZ-treated patients

We have shown that EGF and NTN4 contribute to resistance of cell senescence induced by DNA damage. The co-expression relationship of EGFR/NTN4 prompted us to further explore the effect of EGFR/NTN4 co-expression in GBM patients. We extracted RNA sequencing data and clinical information for 92 TMZ-treated GBM patients from The Cancer Genome Atlas Research Network (TCGA)-glioblastoma dataset [51]. To visualize the relationship among EGFR, NTN4, and patient survival, we constructed a 3D axes plot, with EGFR expression, NTN4 expression, and patient survival on the X, Y, and Z, axes, respectively. In this plot, although EGFR and NTN4 expression did not significantly correlate with poor patient survival (Fig. 4a & b, up-left), co-expression of EGFR and NTN4 did correlate with poor patient survival (Fig. 4c, up-left). To validate the significance of this trend, we selected the 20% of patients with either the highest or lowest expression of target genes and assessed the difference in patient survival between the two groups (Fig. 4a, b, c, right-left). Kaplan-Meier analysis showed that neither EGFR nor NTN4 expression significantly correlated with patient survival after TMZ treatment, while co-expression of EGFR/NTN4 predicts poor patient survival (Fig. 4a, b, c, lower panels). To exclude a random effect of EGFR/NTN4 expression, we analyzed NTN1 and co-expression of EGFR/NTN1 as a control, and observed no correlation with patient survival. Lastly, we generated a violin plot to summarize the comparison of patient survival among all the groups mentioned above, and only the high co-expression of EGFR/NTN4 group showed a condensed low survival time (Fig. 4e).

## Discussion

In this study, we report a cooperative resistance mechanism in GBM cells involving NTN4 and EGFR following DNA-damage induction. We observed the EGF/EGFR signaling pathway as an upstream regulator of NTN4. Activating mutations or amplification of EGFR are observed in 45–57.4% of glioblastomas [52, 53], and frequently occur in the “Classical” subgroup of glioblastomas [51, 54, 55]. Cells with EGFR gene amplification preferentially move to the infiltrating edges of glioblastoma, indicating that EGFR signaling confers a special advantage for GBM progression and migration [56]. Laminin-related integrins, ITGA6 and ITGB4, are co-expressed with different growth factors, especially ones involved with EGF/EGFR signaling [27–29, 47]. Interestingly, a potential integrin-associated protein, netrin-4, is also highly expressed at the invading edge of glioma. By bioinformatic analysis and the EGF stimulation experiment, we confirmed that EGF/EGFR contribute to NTN4 expression.

Using single cell RNA sequencing data from glioblastoma, we enriched for genes co-expressed with EGFR, and further explored the GO bioprocesses and related pathways of EGFR. We found that the gene sets co-expressed with EGFR are involved in various biological functions and pathways, including cell differentiation in GBM, ECM organization, the caspase related pathway, and Notch signaling. These findings are consistent with previous studies [57–60].

Via this analysis and the experiment above, we found that EGF/EGFR regulates NTN4 expression; and that EGFR possibly modulates caspase activation via netrin/netrin receptors [61, 62]. Thus, EGF/EGFR signaling may regulate netrin-related pathways to protect cells from DNA damage. H_2_O_2_ or TMZ can trigger DNA damage in GBM cells. Using these two models, we provide in vitro evidence that either EGF or NTN4 is sufficient to attenuate GBM cell senescence induced by DNA-damage. Interestingly, in vitro co-administration of EGF and NTN4 did not show an additive effect on resistance to DNA damage. These results indicate that EGF/EGFR attenuate GBM cell senescence induced by DNA damage, at least partially via the expression of NTN4.

ERK and AKT pathways are essential in mediating the protective role of EGF/EGFR against DNA damage [33–35]. Our results show that EGF is able to provide additional stimulation to ERK and AKT upon NTN4 treatment, suggesting that EGF may regulate the stimulatory effect of NTN4 on ERK and AKT. However, NTN4 can modulate mTOR phosphorylation independent of EGF, indicating alternative biological functions in response to NTN4.

As an oncogene, EGFR has long been proposed as a drug target for cancer treatment. Many agents were developed as small molecule tyrosine kinase inhibitors (TKIs) to interfere with EGFR tyrosine kinase activity and its downstream signaling. Although EGFR inhibitors were efficacious on GBM cells in vitro, clinical trials performed to date have been disappointing at targeting EGFR in glioblastoma. Despite the availability of TKIs for a broad spectrum of diseases, none of them demonstrate efficacy for glioblastoma treatment, either as a single agent [37] or in combination with other reagents [63, 64] or radiation [65, 66]. In Phase I/II trials, EGFR inhibitors co-administered with TMZ failed to show any effects [36, 37]. Consistent with the clinical trials, our bioinformatic analysis of TCGA glioblastoma revealed that neither EGFR nor NTN4 predicts significant survival in TMZ-treated patients. This suggests that targeting either EGFR or NTN4 singly cannot improve survival of TMZ-GBM patients. However, Patients with high expression of both EGFR and NTN4 show significantly shortened survival time. This result is not in agreement with the observation that no additive effect of EGFR and NTN4 on resistance was seen in vitro; however, this could be because to two reasons. First, in addition to classical ERK/AKT resistant pathways regulated by EGF/EGFR, NTN4 may also modulate ECM/integrin in vivo [67], which can induce ECM reorganization and mechano-transductions. Thus, after EGF/EGFR stimulation, NTN4-ECM-integrins may contribute to the resistance of DNA damage in GBM patients. Second, netrin molecules can regulate microglia, T cells, endothelial cells and leukocytes [68–72]. All these cells may be located near GBM cells in vivo. A potential intercellular resistance mechanism, initiated by EGF/EGFR and exerted by both EGF and NTN4, may contribute to TMZ-DNA damage resistance in GBM patients.

In summary, combinatorial approaches for targeting the *EGFR/NTN4* axis and inducing DNA damage, for example, by temozolomide, may be beneficial in GBM therapy.

## Conclusions

We find that *EGF/EGFR* regulates and cooperates with NTN4 in DNA damage resistance in GBM. Therefore, this provides a potential therapeutic target to the EGFR/NTN4 axis for GBM therapy.

## Abbreviations

ECM: extracellular matrix
EGF/EGFR: Epidermal growth factor/Epidermal growth factor receptor
GBM: Glioblastoma multiforme
ITG: integrin
NTN4: Netrin-4
TMZ: Temozolomide
